# Global trends in refractive disorders from 1990 to 2021: insights from the global burden of disease study and predictive modeling

**DOI:** 10.3389/fpubh.2025.1449607

**Published:** 2025-03-26

**Authors:** Lihong Huang, Dazheng Zhang, Ming Liu

**Affiliations:** ^1^Dujiangyan Medical Centre, Chengdu, China; ^2^Chengdu University of Traditional Chinese Medicine, Chengdu, China; ^3^Dujiangyan Juvenile Myopia Prevention and Treatment Center, Chengdu, China; ^4^Chengdu Women's and Children's Medical Center, Chengdu, China

**Keywords:** refraction disorder, global burden of disease study, prevalence, disability-adjusted life years, age-related

## Abstract

**Purpose:**

This study aims to provide global, regional, and national estimates of the refractive disorders-related burden between 1990 and 2021, stratified by subtypes.

**Design:**

A retrospective analysis was conducted using aggregated data.

**Methods:**

Utilizing data from the Global Burden of Diseases, Injuries, and Risk Factors Study 2021 (GBD 2021), this population-based study analyzed the burden of refractive disorders from 1990 to 2021. Estimated annual percentage changes (EAPCs) were calculated to quantify temporal trends in age-standardized rates of refractive disorder prevalence and disability-adjusted life-years (DALYs), stratified by age, sex, region, and country. The associations between EAPCs in age-standardized rates and socio-demographic index (SDI) were also examined. Using comprehensive data, we conducted predictive analyses with the Age-Period-Cohort (APC) and Autoregressive Integrated Moving Average (ARIMA) models to forecast disease prevalence and Disability-Adjusted Life Years (DALYs) for informing future public health strategies.

**Results:**

Globally, the age-standardized rates of refractive disorders per 100,000 population decreased in all age groups from 1990 to 2021. Specifically, prevalence rates declined from 2,053.56 (95% uncertainty interval [UI]: 1,835.31–2,275.8) to 1919.66 (95% UI: 1,715.24–2,135.28, EAPC: −0.24), while DALYs reduced from 88.04 (95% UI: 62.19–125.15) to 79.11 (95% UI: 54.94–114.14, EAPC: −0.36). These reductions were primarily driven by a decline in refractive disorders. Notably, both the prevalence and DALYs associated with refractive disorders decreased significantly in the 60 to 64 age group. In low and low-middle SDI countries, there was a significant reduction in both prevalence and DALYs, while high and middle-high SDI countries experienced an increase in these metrics. Benin exhibited the largest increase in prevalence and burden, while India had the most significant decrease. There are notable discrepancies between countries and regions compared to GBD estimates, indicating potential underestimations of refractive disorder prevalence and burden. The APC model details age-specific trends and cohort effects, while the ARIMA model offers strong predictions from historical data. Both models underscore fluctuating disease burdens, stressing the importance of adaptive health policies to meet future healthcare needs.

**Conclusion:**

Over the past three decades, global efforts have significantly alleviated the burden of refractive errors. However, substantial disparities persist across different types of impairment, age groups, and countries' Socio-Demographic Index (SDI). With the conclusion of the COVID-19 pandemic, it is crucial to expand eye care services, particularly in enhancing screening coverage and quality control. This study underscores the importance of addressing diverse population needs and fostering regional cooperation to improve eye health outcomes, providing a comprehensive strategy for future public health initiatives.

## 1 Introduction

Studies reveal that ~42% of global visual impairments are attributed to refractive disorders (1). Refractive disorders refer to common eye conditions in which the eye cannot properly focus light on the retina, leading to blurred vision. This results from an irregular shape or length of the eye, or abnormalities in the cornea or lens. The primary types of refractive disorders include Myopia, Hyperopia and Astigmatism. Most refractive disorders can be effectively corrected with glasses, contact lenses, or refractive surgery, restoring clear vision and enhancing well-being. Refractive disorders are the leading cause of visual impairment globally. When left uncorrected, they can significantly affect quality of life, academic performance, productivity, and social engagement, imposing a substantial economic burden on societies. By 2050, it is estimated that 4.758 billion individuals worldwide will experience myopia, with 938 million suffering from high myopia. China, the world's most populous nation, contributes significantly to these figures due to its high prevalence of refractive errors in recent decades (2). Refractive disorders have substantial psychological, educational, and socioeconomic impacts, not only in childhood but also in adulthood (3). Visual impairment accounts for one-third of the global economic cost associated with preventing and treating visual impairment and blindness.

Studies have shown (4) a significant link between visual impairment and mental disorders such as depression (5), anxiety (6) and cognitive impairment (7) and so on. These issues are major public health concerns for the older adult, leading to distress, family disruption, disability, worsening of other conditions, and increased mortality (8). As visual impairment and mental disorders both increase with age, their coexistence exacerbates societal burdens through functional loss, loneliness, and higher mortality (9). Early identification and treatment of these conditions are crucial for improving older adult well-being. This underscores the need for integrated health strategies to address both visual and mental health challenges in aging populations.

A systematic review and meta-analysis based on the Global Burden of Diseases, Injuries, and Risk Factors Study (GBD) 1990–2019 reported a decreasing burden of vision loss in the general population. Further, among children under 14 years a downward trend in disability-adjusted life years (DALYs) for refractive disorders globally (10). Similarly, studies from global, regional, and national trends in refractive disorder prevalence, DALYs, and time trends from 1990 to 2019 illustrate that refractive disorders are underestimated (11). However, there remains a global lack of comprehensive understanding regarding the burden of disease and incidence data related to visual impairment, and predictive models for DALYs associated with these conditions are still insufficient. Additionally, there is a shortage of recent reports on the burden of different types of vision loss and their relationship with the Sociodemographic Index (SDI).

GBD estimates disease burden by combining years lived with disability (YLDs) and years of life lost (YLLs) into DALYs, where one DALY represents the loss of one healthy year of life (12). In collaboration with the GBD, the database has been extensively updated and improved with further available data until 2021, allowing for a more precise estimation of the vision loss burden (12). This is particularly relevant in light of concerns regarding possible overdiagnosis of refractive disorders over the years.

To address these gaps, the present study aimed to describe the prevalence rates and DALYs of blindness and visual loss, as well as their trends between 1990 and 2021, at the global, regional, national levels, and by disease type. The first objective was to present the prevalence and burden of refractive disorders by country/region and sex for each year in the period 1990–2021, as estimated by the GBD. Additionally, given the influence of GBD data on research and policies, we aimed to develop predictive models and critically assess the extent to which the 2019 GBD may have miscalculated the prevalence and burden of refractive disorders.

## 2 Methods

### 2.1 GBD data: overview and case definition and data collection

#### 2.1.1 Overview and data sources

The GBD 2021 Results Database, accessible through the GBD Collaborative Network website (http://ghdx.healthdata.org), comprises data The following data regarding refraction disorders were acquired from the Global Health Data Exchange (http://ghdx.healthdata.org/gbd-results-tool). This study employs the GBD 2021 definition of refractive disease, specifically adhering to the Snellen chart standard. In the GBD 2021, disease prevalence was estimated by incorporating data on past-year prevalence, while lifetime prevalence was not considered due to potential risks associated with recall bias. The GBD 2021 team conducted a systematic review of PsycINFO, Embase, and PubMed databases, retrieving data on disease prevalence up to October 10th, 2021. The included sources comprised surveys employing probability sampling to procure a representative sample of the general population (13). Surveys relying on non-probabilistic sampling or focusing on population subgroups were excluded. Acceptable definitions of refraction disorder adhered to the criteria outlined in Basic Ophthalmology or the Overview of refractive disorders.

From this study, data from global incidence, prevalence, years lived with disability (YLDs), DALYs, and healthy life expectancy (HALE) for 371 diseases and injuries in 204 countries and territories and 811 subnational locations, 1990–2021 (14). DALYs was calculated by multiplying the number of incident cases of refraction disorder in the population by a disability weight specific to that condition and the average duration of the case until remission or death (15).

DALYs, a comprehensive measure of disease burden, were calculated for refractive disorders by multiplying the number of incident cases by the condition-specific disability weight and the average duration of the condition until remission or death. While DALYs generally comprise the sum of YLDs and years of life lost (YLLs) due to premature death, refractive disorders are not associated with premature mortality. Consequently, DALY estimates for refractive disorders are equivalent to YLDs.

#### 2.1.2 Prediction model

To assess global trends in the prevalence rates and DALYs of refraction disorder, we calculated age-specific rates and their average annual percentage changes (EAPCs) using linear regression with logarithm-transformed rates as the dependent variable and year as the independent variable (14). The EAPC, a weighted average of annual percentage changes (APCs), provides a concise summary of trends over a specified interval, enabling the description of average APCs across multiple years using a single value. Prevalent case counts, DALYs, and related rates were extracted directly from the GBD 2021 dataset, with all rates reported per 100,000 population. The 95% uncertainty intervals (UIs) were derived from the 25th and 975th percentiles of the ordered 1,000 estimates, adhering to the GBD algorithm (14).

#### 2.1.3 Statistical analysis

To address study heterogeneity encompassing sample size and selection, examination rates, and diagnostic criteria, the GBD estimation tool DisMod quantified the between-study heterogeneity—the variance unaccounted for by fixed effects or geographical random effects—and incorporated the associated uncertainty. Additionally, the GBD 2021 introduced the SDI for each country, a composite indicator reflecting social and economic conditions that influence health outcomes. This index is calculated as the geometric mean ranging from 0 to 1, encompassing indices such as the total fertility rate among individuals younger than 25 years, mean years of education for those 15 years and older, and lag distributed income per capita, where 0 signifies the lowest income level and highest fertility rate (14). Notably, the GBD 2021 did not incorporate adjustments for bias from small community samples or two covariates accounting for estimates without informant agreement (e.g., parent-child) or diagnostic impairment, as compared to GBD 2019. This omission was justified by expert consultations highlighting uncertainties surrounding systematic biases across survey methodologies.

We focused on the EAPCs between 1990 and 2021, employing linear regression analysis to identify temporal trends and fit the simplest model possible to the data by connecting multiple line segments on a logarithmic scale (16). The final model was implemented in Linear 4.9.0.0 software (National Cancer Institute, Information Management Services, Inc, United States). All statistical analyses were conducted using GraphPad Prism (version 8.0), RStudio software (version 1.4.1106), and the Linear Regression Program (version 4.9.0.0).

### 2.2 SDI

The SDI, an aggregative metric, serves as a comprehensive gauge of a country or region's development. It incorporates data on the total fertility rate among females under 25, the average educational level of females aged 15 and above, and per capita income (11). Based on the SDI index, the GBD 2021 database stratifies the world into five distinct regions: low-SDI (0–0.45), low-middle-SDI (0.45–0.61), middle-SDI (0.61–0.69), high-middle-SDI (0.69–0.81), and high-SDI (0.81–1).

### 2.3 Age-standardized rate

ASR, a pivotal indicator in epidemiology, is particularly useful when comparing groups with varying age compositions. Direct comparisons of crude rates may introduce bias as they fail to account for differences in age structure. Standardization is thus necessary to eliminate this confounding factor. The age-standardized rate per 100,000 population is calculated by summing the products of age-specific rates and the corresponding number of cases within each age subgroup of a selected reference standard population, then dividing this sum by the total of the standard population weights (14). In this study, we employed the GBD world population as the reference standard to calculate ASRs, which were subsequently used to quantify the burden of two types of depression and assess the trends in DALYs.

### 2.4 Age-period-cohort modeling analysis of incidence data

The APC model serves as a robust statistical approach for extracting and elucidating illness patterns, while evaluating the distinct contributions of age, period, and cohort effects on outcomes. In this study, our focus lies primarily on the following estimable functions: Net drift, which captures the overall annual percentage change; local drifts, reflecting annual percentage changes specific to each age group; the longitudinal age curve, illustrating the fitted age-specific rates for the reference cohort after accounting for period deviations (17).

To mitigate the identification challenge posed by the linear dependencies between age, period, and cohort, we employed the intrinsic estimator (IE) method, inherent to the APC model. This approach enables us to overcome the limitation of unpredictable model parameters. For a detailed methodological discussion, we refer to previous literature (17). The APC analysis for this study utilized the APC Web Tool (http://analysistools.nci.nih.gov/apc/) provided by the National Cancer Institute. All visualizations were generated using the R statistical program (version 4.0.3).

### 2.5 Autoregressive integrated moving average

The ARIMA model, formally known as the autoregressive integrated moving average model, is a widely adopted technique for time series forecasting analysis. In the ARIMA (*p, d, q*) specification, “AR” stands for “autoregressive,” with “p” indicating the number of autoregressive terms. Similarly, “MA” stands for “moving average,” with “*q*” designating the number of terms in the moving average component. The “*d*” parameter represents the number of differences (or order) applied to the data to achieve stationarity (18).

In this study, we employed the ARIMA model to analyze the trend in disease burden, quantified by DALYs, and further projected the burden of a specific neglected refractive disorder from 2020 to 2030. Additionally, we performed a descriptive analysis to assess the prevalence of this refractive disorder and its associated DALYs in China for the years 1990 and 2021. The sex and age distribution of the refractive disorder in 2021 was also investigated. All analyses and data visualization were conducted using R version 4.2.1 (19).

### 2.6 Critical re-analysis of GBD data from 2019

We adopted a random-effects model, weighted by the inverse of variance, to calculate the pooled prevalence of refractive disorders from these selected studies. To ensure comparability with previous systematic reviews and meta-analyses, we included studies spanning from 1990 to 2021, aligning with GBD and encompassing various geographical regions. Therefore, we compared the data from two relevant papers in 2019, One is from Global, regional, and national prevalence, disability adjusted life years, and time trends for refraction disorders; and time trends for refraction disorders (11); the other comes from Gender Disparities in the Global Burden of gender disparities Refractive Disorders in Children (10). Furthermore, to assess the impact of socio-economic status on the prevalence of refractive disorders in 2021, we performed a meta-regression analysis, utilizing the GBD socio-demographic index (SDI), which accounts for income per capita, educational attainment, and total fertility rate among women under 25 years old.

## 3 Results

### 3.1 Global analysis: description and trend

This section reports the age-standardized prevalence and DALYs for refractive disorders from 1990 to 2021, with data presented every 5 years. Supplementary Table 1 presents the trends in prevalence (per 100,000 population) and DALYs (per 100,000 population) for refractive disorders across all ages from 1990 to 2021. Globally, the prevalence and DALYs rates declined from 1990 to 2021, with estimated annual percent changes (EAPC) of −0.24 (95% CI: −0.27 to −0.21) and −0.36 (95% CI: −0.4 to −0.32), respectively. Notably, while DALYs increased from 1990 to 2000, they declined after 2,000. However, the DALYs and prevalence of refractive disorders continued to increase annually, with an EAPC of 0.09.

### 3.2 Sex and age

#### 3.2.1 Age group disparities

**Figure 1:**
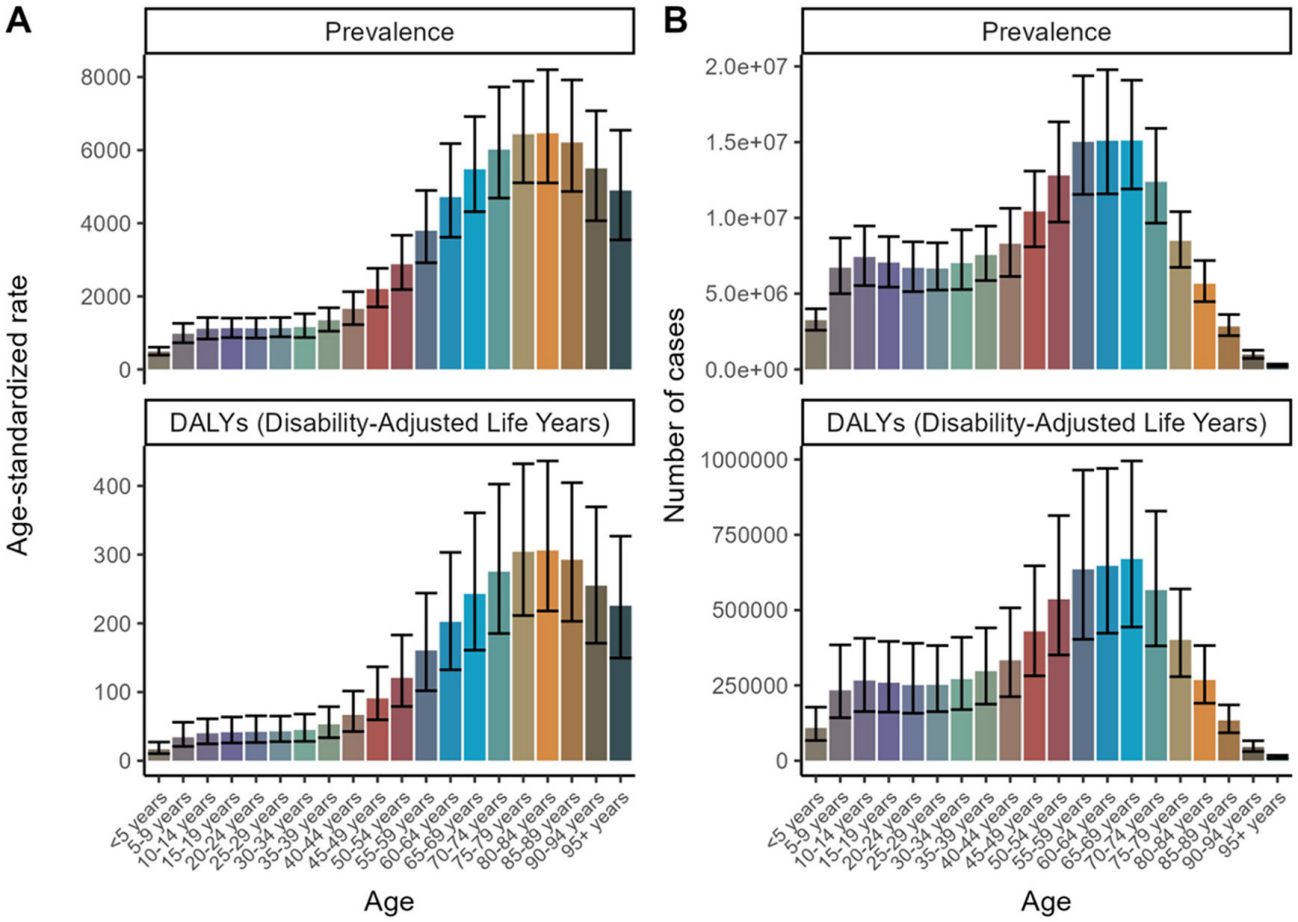
Age Trends for 2021 to forecast future trends in disease prevalence and Disability-Adjusted Life Years (DALYs). **(A)** Age-standardized rate. **(B)** Number of cases.

**Figure 2:**
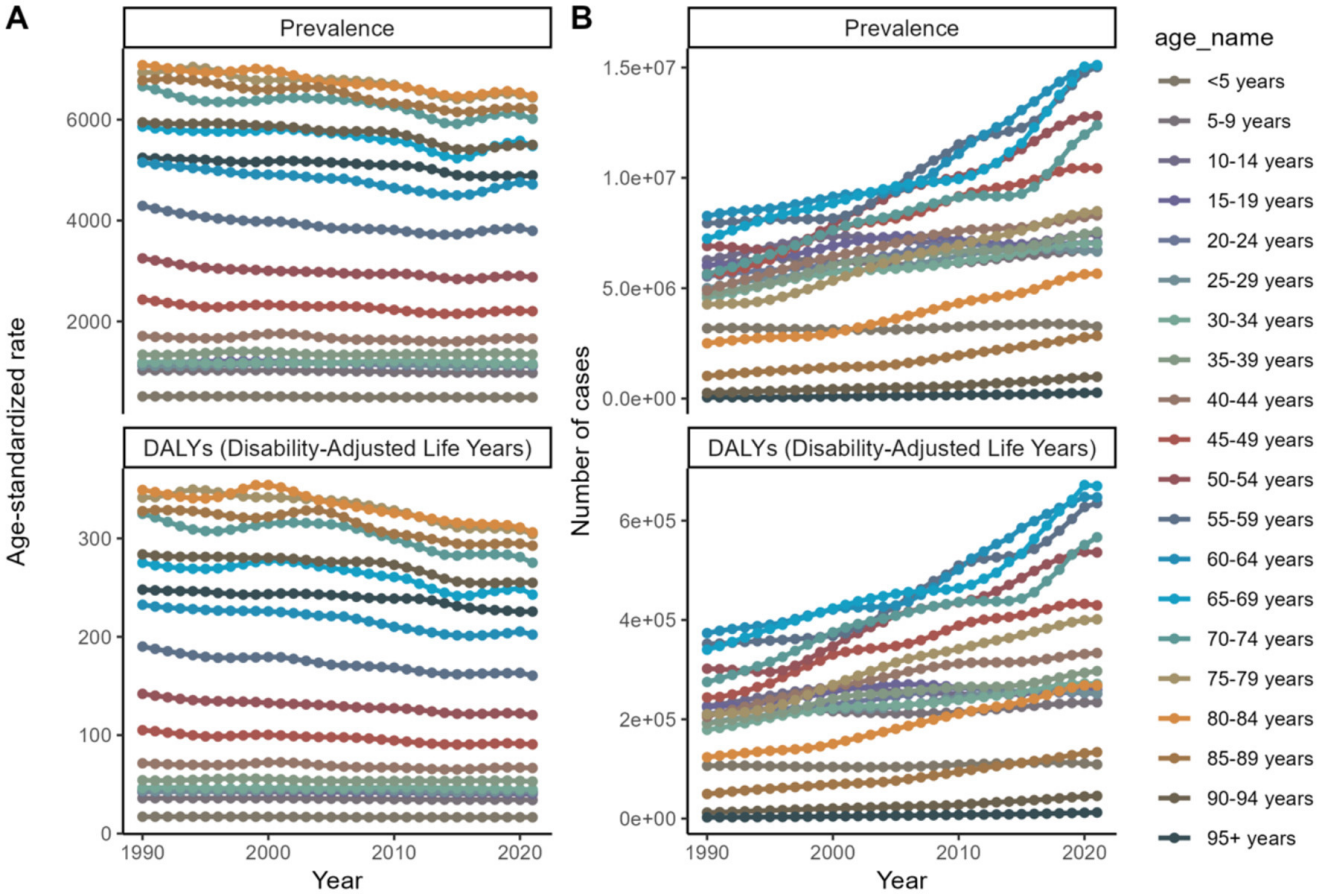
Age trends from 1990 to 2021 to forecast future trends in disease prevalence and Disability-Adjusted Life Years (DALYs). **(A)** Age-standardized rate. **(B)** Number of cases.

Younger age groups (<5 years) consistently exhibit higher age-standardized rates for both prevalence and DALYs, signifying a disproportionately heavier disease burden. Conversely, older age groups (65+ years) display a marked escalation in case counts and DALYs, reflecting the impact of an aging population and the commensurate augmentation in disease burden within these demographic cohorts. Age-adjusted male-to-female ratios for refractive disorder prevalence and DALYs demonstrate a significant increase with advancing age. Over a span of 30 years, a general upward trend in both case counts and DALYs is observed across all age brackets, indicative of a progressively increasing global burden of disease. Despite the upward trend in case numbers and DALYs, the age-standardized rates for the majority of age groups remain largely unchanged. This stability points to population growth and aging as primary drivers behind the rising case counts and DALYs (Figures 1, 2 and Appendix 1).

#### 3.2.2 Sex group disparities

**Figure 3:**
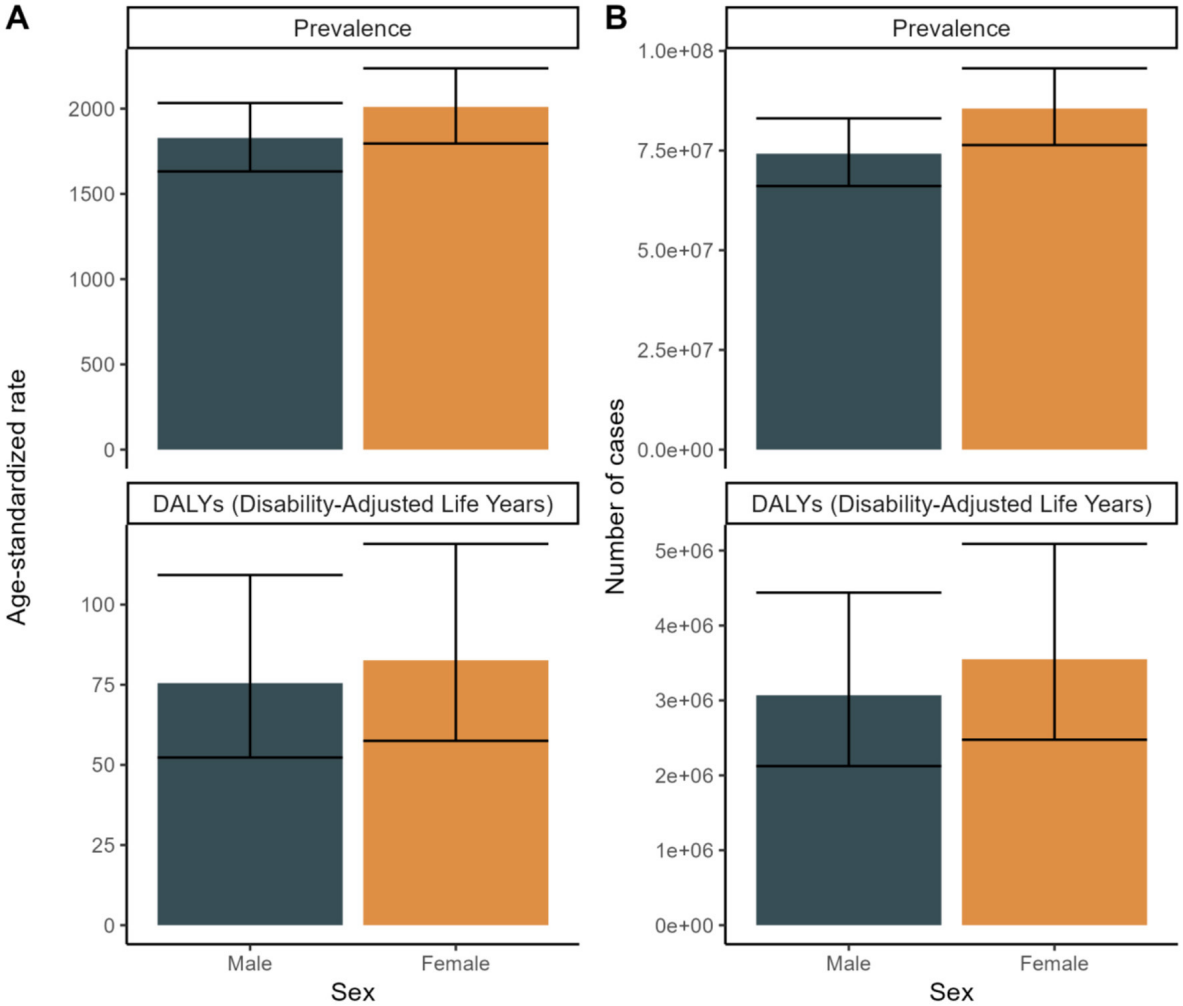
Sex Trends for 2021 to forecast future trends in disease prevalence and Disability-Adjusted Life Years (DALYs). **(A)** Age-standardized rate. **(B)** Number of cases.

**Figure 4:**
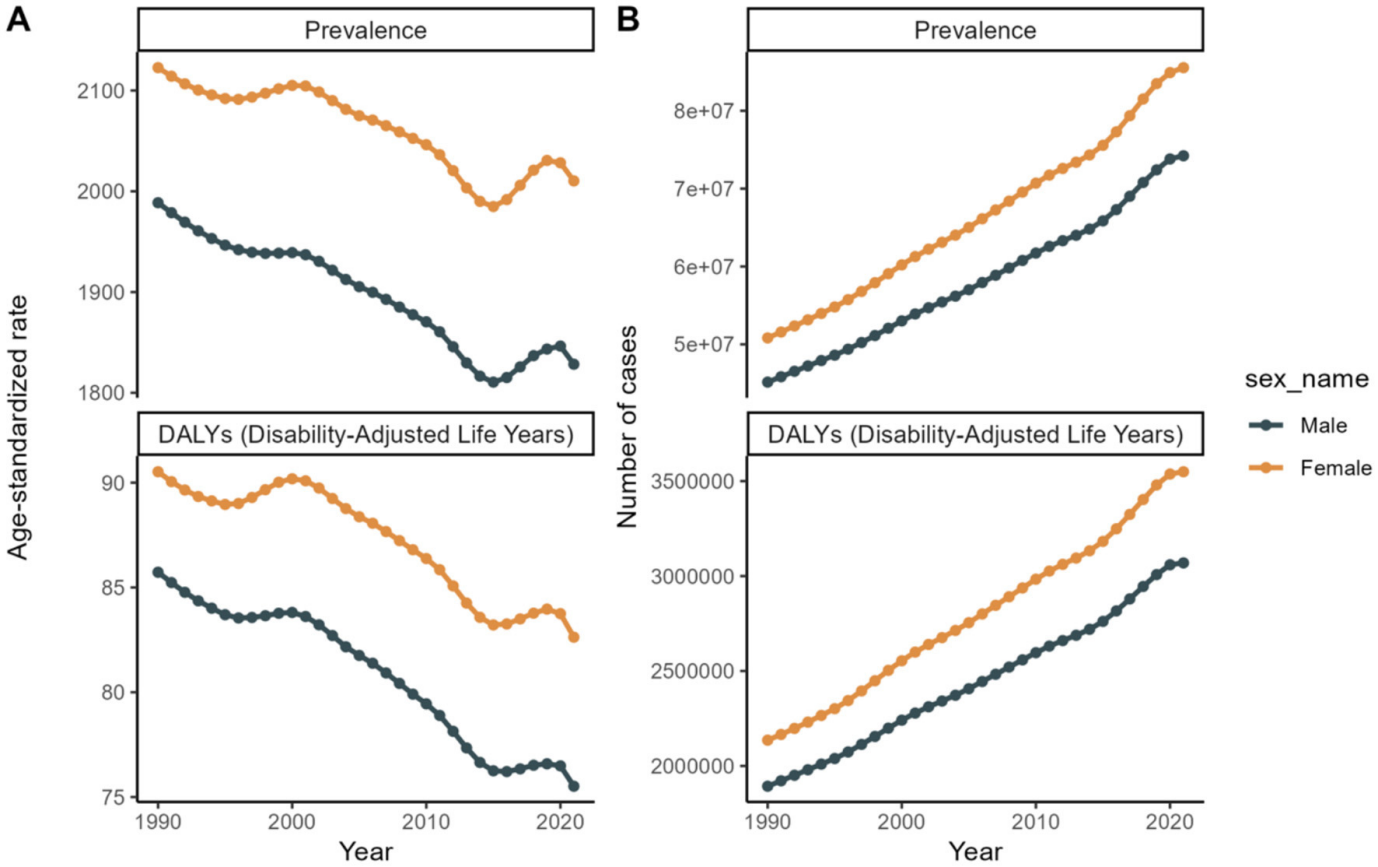
Sex trends from 1990 to 2021 to forecast future trends in disease prevalence and Disability-Adjusted Life Years (DALYs). **(A)** Age-standardized rate. **(B)** Number of cases.

In terms of sex, the global prevalence and DALYs rates were consistently lower among males compared to females, indicating a consistent advantage for women. An analysis of prevalence and DALYs across age groups for both sexes reveals distinct patterns. Specifically, between the ages of 80 and 84, both sexes exhibit a rise in prevalence and DALYs, with females exhibiting higher figures. GBD 2021 study estimates that by 2040, the worldwide prevalence of refractive disorders will surpass 21.12 million women and 19.14 million men. Between 1990 and 2021, the prevalence of refractive disorders rose from 9.5 million (95%UI = 1,835.31–2,275.8) to 15.9 million (95%UI = 1,715.24–2,135.28), representing a relative increase. Simultaneously, the raw DALYs associated with refractive disorders increased by 36% from 1990 to 2021 (Figures 3, 4 and Appendix 1).

### 3.3 Country and region

**Figure 5:**
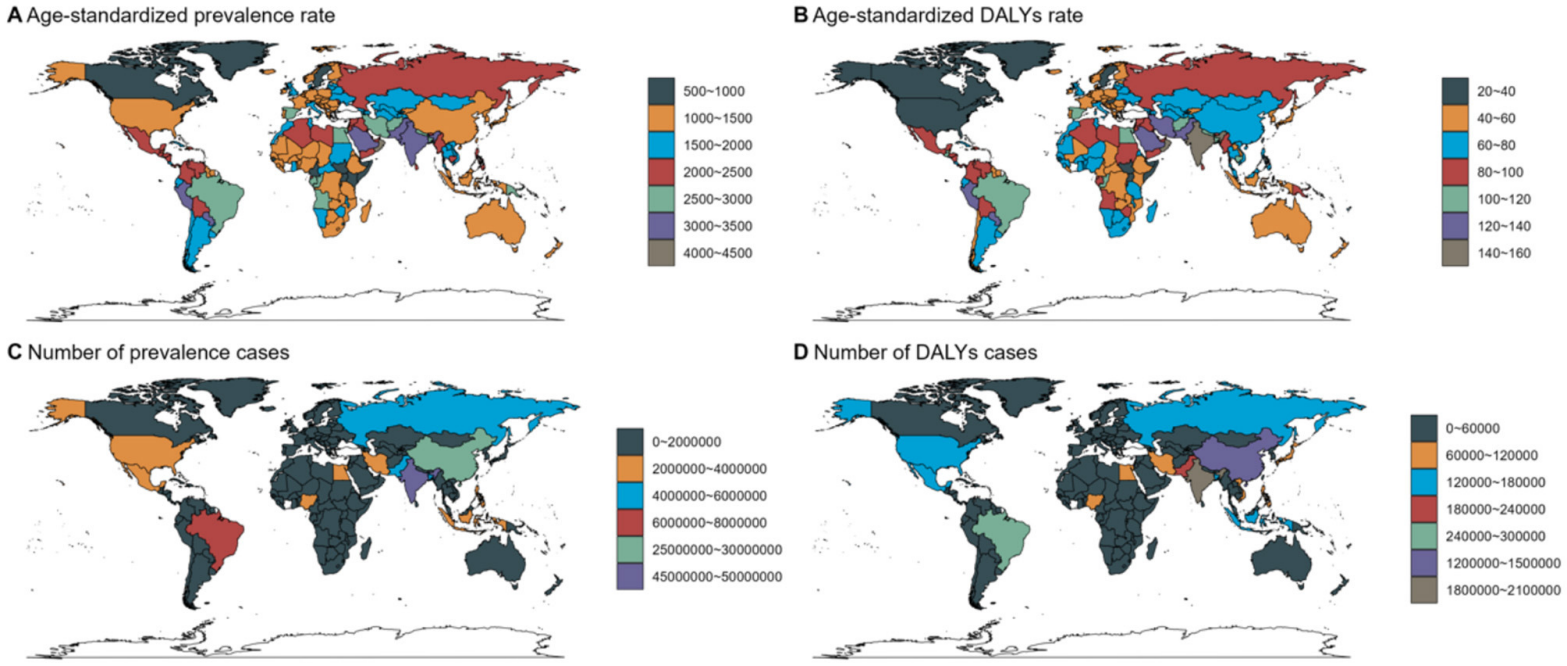
Country Trends for 2021: age standardized prevalence and DALY rates. Age-standardized **(A, B)** prevalence **(C, D)** DALY rates (per 100 000) by location, both sexes combined, 2019.

**Figure 6:**
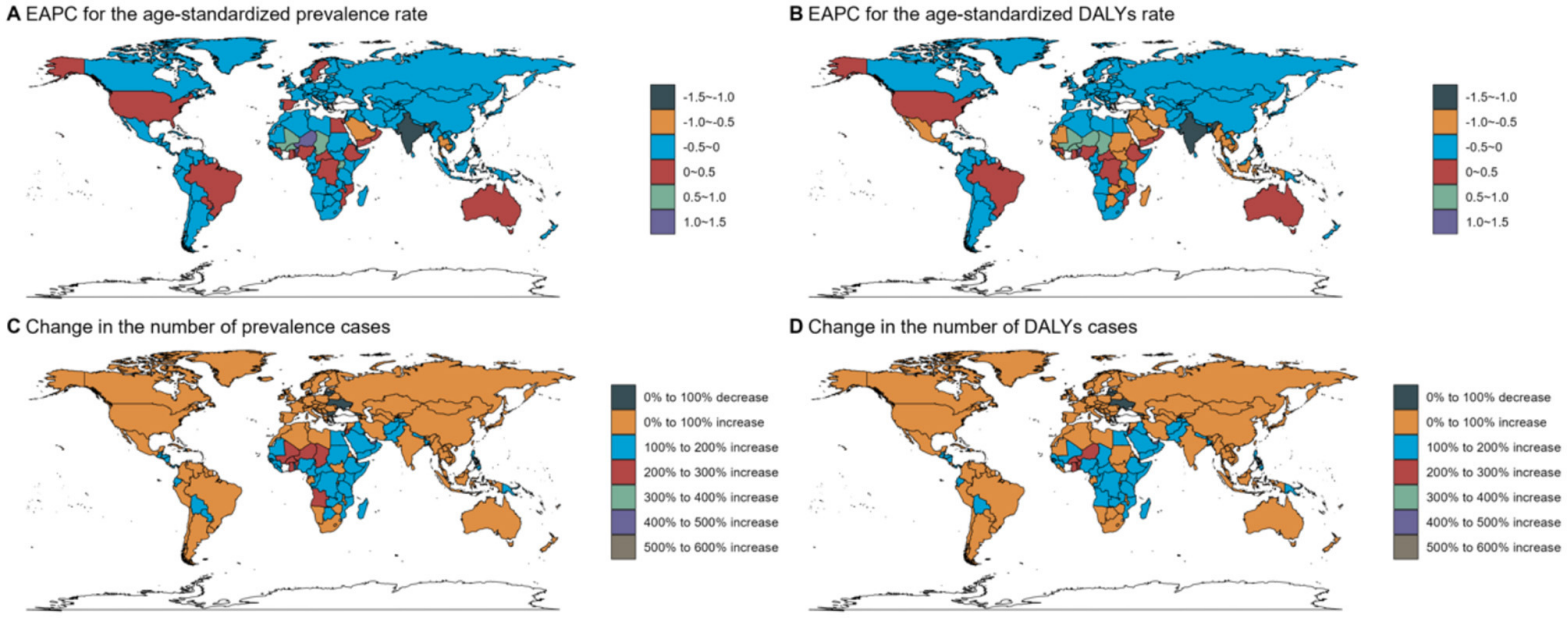
Country trends from 1990 to 2021: age standardized prevalence and DALY rates. Age-standardized **(A, B)** prevalence **(C, D)** DALY rates (per 100 000) by location, both sexes combined, 2019. DALY disability-adjusted life years.

**Figure 7:**
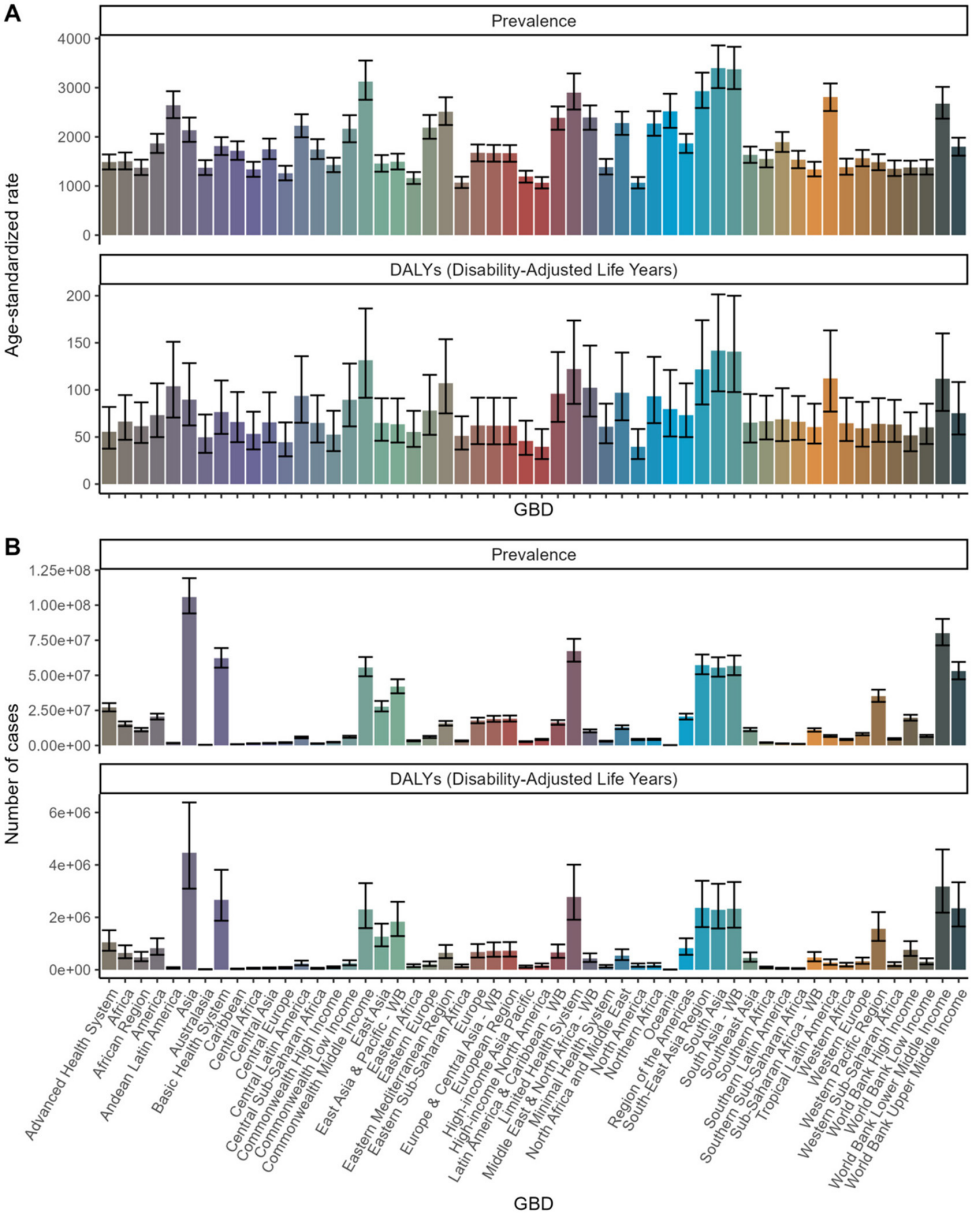
Global Burden of Disease (GBD) Analysis: Prevalence and DALYs across Health Systems and Regions. **(A)** Age-standardized rate. **(B)** Number of cases.

Refractive disorders exhibited varying prevalence and disability figures across different countries and GBD regions. Overall, the prevalence and DALYs rates attributed to refractive disorders decreased in most regions and countries (Figures 5, 6). In 1990, Asia and the Pacific had the highest DALY rates (69.3 per 100,000 people), while in 2021, the Caribbean region recorded the highest DALY rates (63.55 per 100,000 people). Conversely, Oceania had the lowest DALYs in 1990 (83.02/100,000 population) and Oceania's World Bank Lower Middle-Income category recorded the lowest DALY rate in 2021 (79.66/100,000 population). Australasia stands out with the highest age-adjusted prevalence of refractive disorders, surpassing other regions (Figure 7 and Appendix 2).

Notably, the prevalence of refractive disorders increased in certain East Asian countries, while the DALY rates increased in high-income countries in North America. In 1990, the top three countries with the highest prevalence rates were India (4,778.76/100,000), China (1,567.67/100,000), and the Russian Federation (2,389.76/100,000). By 2021, India (3,495.7/100,000), China (1,467.89/100,000), and Brazil (2,795.57/100,000) topped the list, with China maintaining its position. In 2021, Russia, with a prevalence rate of 2311.39/100,000, ranked sixth (Appendix 2).

The EAPCs in prevalence between 1990 and 2021 were below zero in several countries and regions, including India (−1.11) and China (−0.23). The DALY burden ratio decreased in the Caribbean (−0.68) and Southern Africa (−0.93). Conversely, there was a significant increase in the prevalence of refractive disorders in Brazil (0.19), the United States of America (0.08), and Nigeria (0.04). Between 1990 and 2021, the largest decreases in DALY rates were observed in Eastern Europe (0.23), the African Region (0.18), and South Asia (0.11). During this period, Benin experienced the largest increase in prevalence and burden, while India saw the largest decrease (Appendix 2).

**Figure 8:**
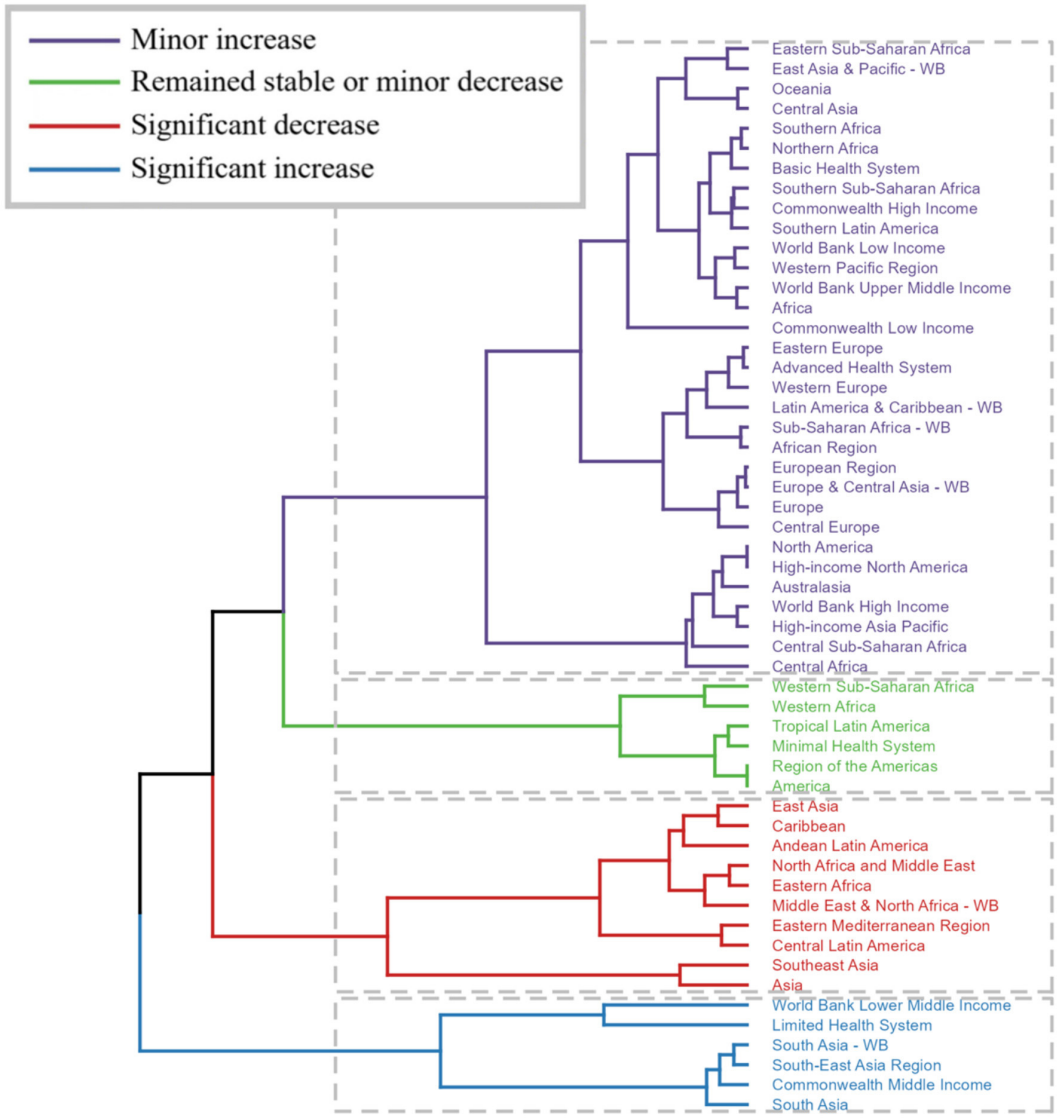
Trends in Global Burden of Disease (GBD): Cluster analysis, estimated annual percent change data combining incidence and deaths. (Regions exhibiting significant increases (depicted in blue) may require urgent intervention and resource allocation. Regions with marked decreases (depicted in red) might provide insights into successful health strategies that could be replicated in other areas. Regions with minor changes (depicted in black and green) indicate stability but might still necessitate attention to prevent future increases.).

The dendrogram offers an additional layer of analysis, clustering regions based on their changes in disease burden. This approach aids in identifying patterns and potential underlying causes for these variations. Regions exhibiting significant increases (depicted in blue) may require urgent intervention and resource allocation. Regions with marked decreases (depicted in red) might provide insights into successful health strategies that could be replicated in other areas. Regions with minor changes (depicted in black and green) indicate stability but might still necessitate attention to prevent future increases (Figure 8).

### 3.4 SDI

#### 3.4.1 Age-standardized rates and case numbers

**Figure 9:**
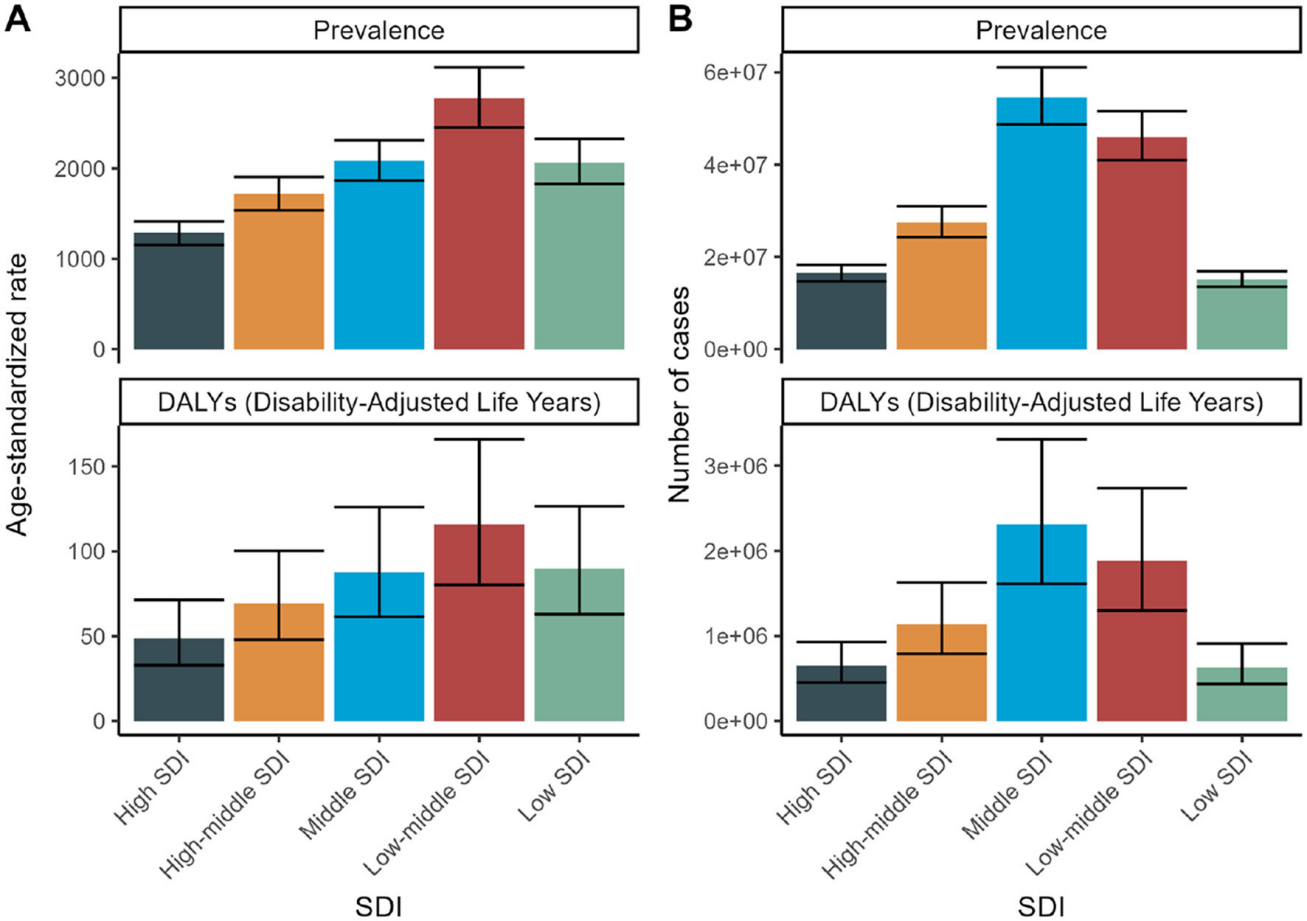
SDI Trends for 2021: Health burden of refraction disorders in SDI regions in 2019. Gender-specific burden in terms of age-standardized DALY rates. **(A)** Age-standardized prevalence of refractive disorders across SDI regions. **(B)** Number of cases of refractive disorders across SDI regions.

Although the age-standardized rates for prevalence and DALYs exhibit a general decline or stability, the absolute number of cases and DALYs has been escalating. This trend suggests that while the individual risk may be diminishing, the overall burden is intensifying due to population expansion and aging. Specifically, the low- and middle-low SDI groups exhibited upward trends in the prevalence rates of refractive disorders from 1990 to 2020, whereas the high SDI group did not display a significant change (Figure 9).

#### 3.4.2 Socioeconomic disparities

High SDI countries demonstrate the lowest prevalence and burden, presumably attributed to improved access to ophthalmic care and corrective interventions. Conversely, low and low-middle SDI countries exhibit the highest prevalence and burden, reflecting constrained access to healthcare and preventive services (Figure 9).

#### 3.4.3 Trends over time

**Figure 10:**
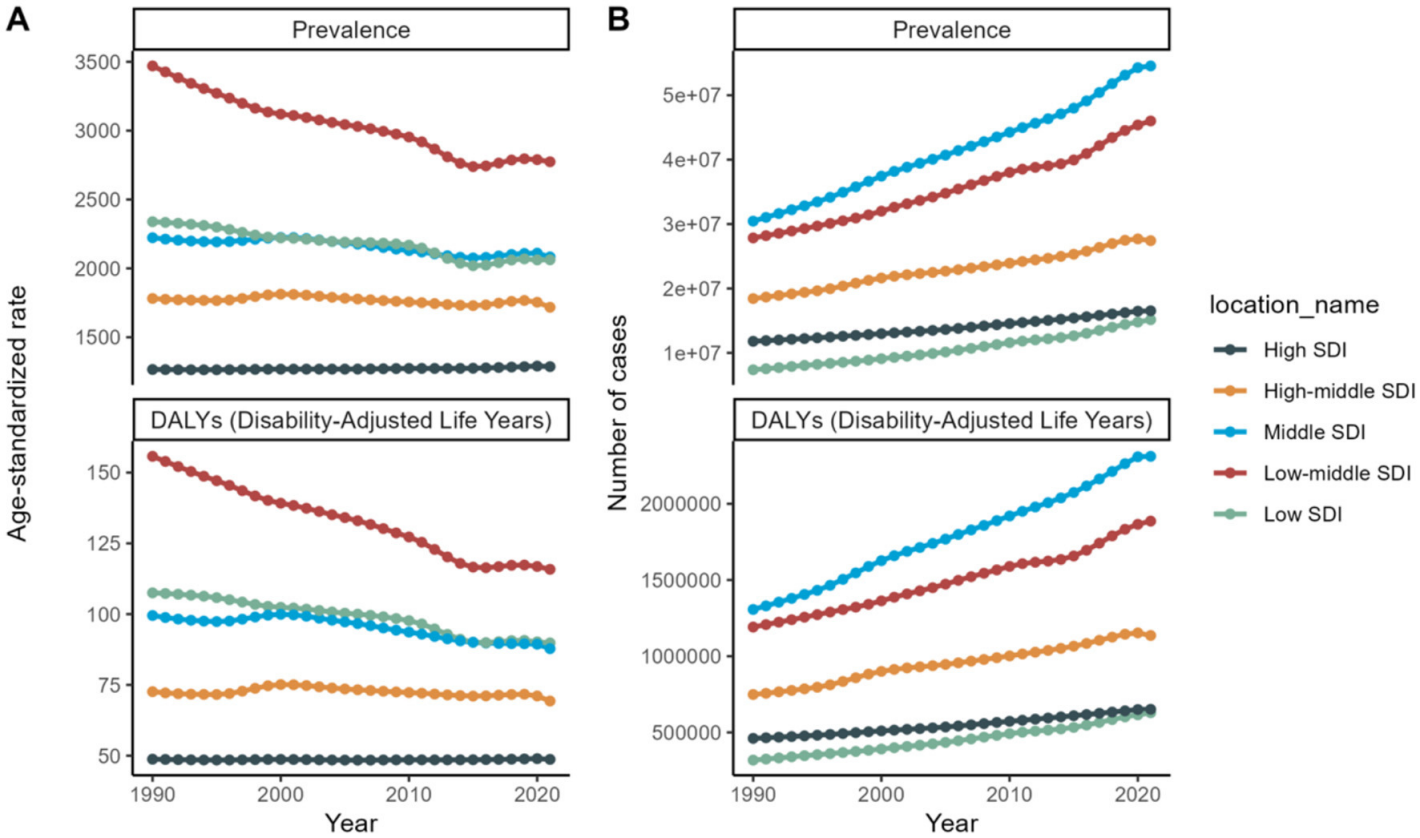
SDI trends from 1990 to 2021: Health burden of refraction disorders in SDI regions in 2019. Gender-specific burden in terms of age-standardized DALY rates. **(A)** Trends in age-standardized prevalence of refractive disorders across SDI regions. **(B)** Trends in number of cases of refractive disorders across SDI regions.

Across all SDI levels, there is a consistent upsurge in the number of cases and DALYs, highlighting the urgency for global health strategies to address the escalating burden of refractive disorders. The data underscores the need for targeted health policies, particularly in low and low-middle SDI regions, to enhance access to ophthalmic care and mitigate the burden of refractive disorders. By comprehending these trends and the underlying socioeconomic factors, policymakers can allocate resources more efficiently and implement interventions to alleviate the global health burden of refractive disorders (Figure 10).

### 3.5 Comparison with the GBD 2019 analysis

A 2019 analysis found that socioeconomic development is closely linked to the prevalence of childhood vision disorders, with girls in lower-income countries bearing a disproportionately higher burden than males (10). Gender-specific health policies are crucial to address these disparities. Despite a global reduction in childhood vision impairment from 1990 to 2019, myopia rates have increased, especially in high-income countries (10). Efforts should target expanding eye care services and improving screening coverage, particularly for vulnerable groups like premature infants in low-income regions and children with limited outdoor activities in high-income regions.

Unlike previous studies, our current study examines all age groups and identifies significant variations in the augmentation of refractive disorders across different countries and regions. These insights may contribute to the development of more comprehensive plans to prevent and manage refractive disorders.

### 3.6 Predictive model analysis: APC and ARIMA

**Figure 11:**
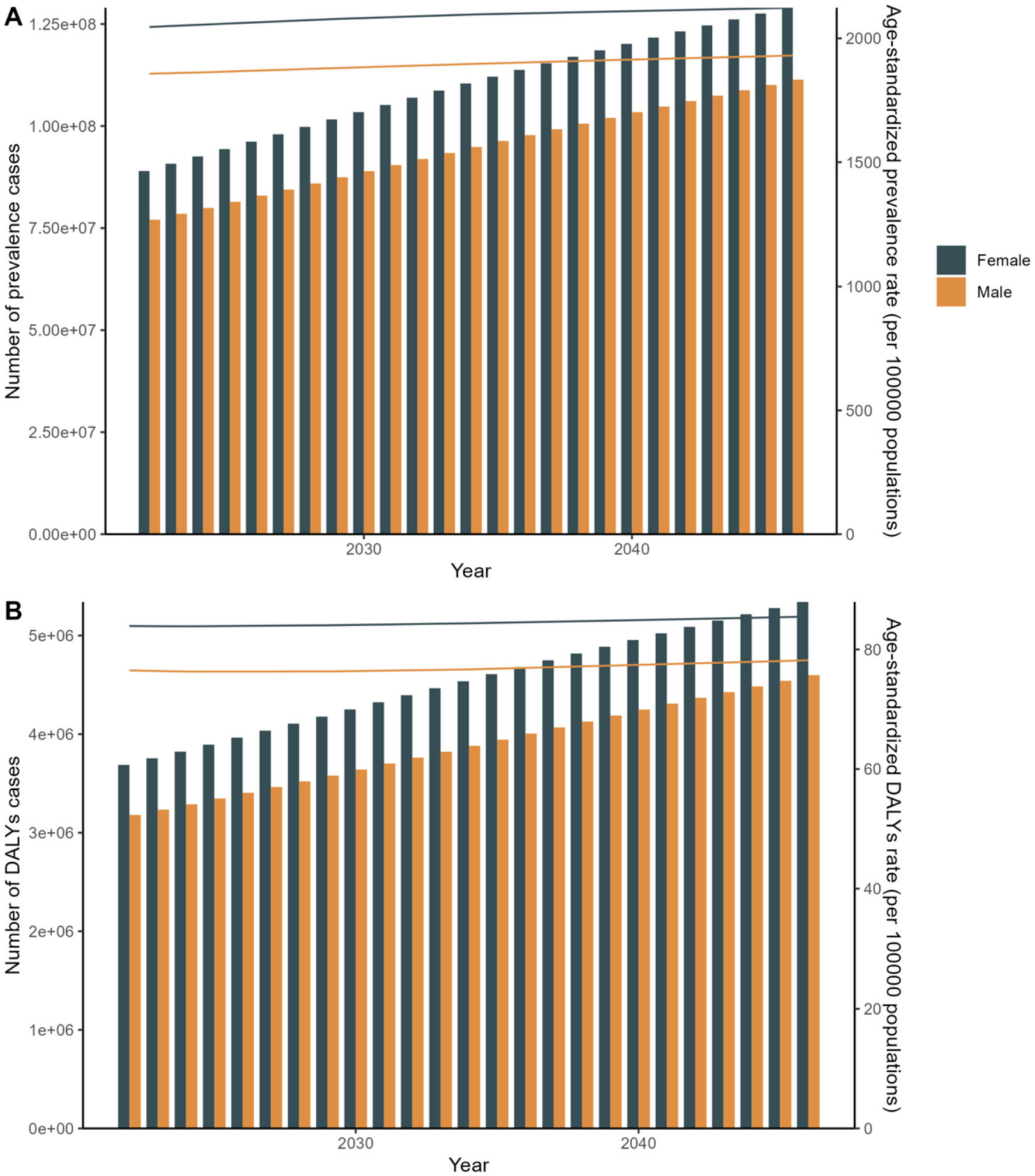
APC: Linear regression analysis of the sex-specific age-standardized incidence rate for refraction disorders from 2030 to 2040. **(A)** Prevalence rate. **(B)** DALYs rate.

Figure 11 depicts the APC analyses of the age-standardized incidence rates for refractive errors (RE) from 1995 to 2046, focusing specifically on female populations. Our findings indicate a significant upward trend in disease incidence over this period. Furthermore, APC analyses were conducted on the age-standardized prevalence and DALYs rates for both sexes.

Disease prevalence and DALYs are on the rise over the next two decades. Chart A shows an increasing trend in disease prevalence and age-standardized prevalence rates for both males and females, with female figures consistently higher than those of males. Chart B illustrates that the number of DALYs cases and age-standardized DALYs rates are also increasing, with females again showing higher figures than males. These trends highlight gender differences in future public health challenges and the growing health burden. APC Model: Focuses on understanding disease trends by decomposing the effects of age, period, and cohort. It provides detailed insights into specific population segments and their changes over time (20). Although, the absolute number of cases and DALYs might show varied trends, age-specific rates and overall trends provide a nuanced understanding of how different age groups and cohorts are affected over time. ARIMA Model: Primarily used for forecasting based on historical data, incorporating patterns of autoregression, differencing, and moving averages to predict future trends (21). Indicates a robust forecast of increasing disease burden in terms of both prevalence and DALYs, highlighting the potential for rising healthcare demands and the need for enhanced medical interventions and policies (Figure 11 and Appendix 3).

**Figure 12:**
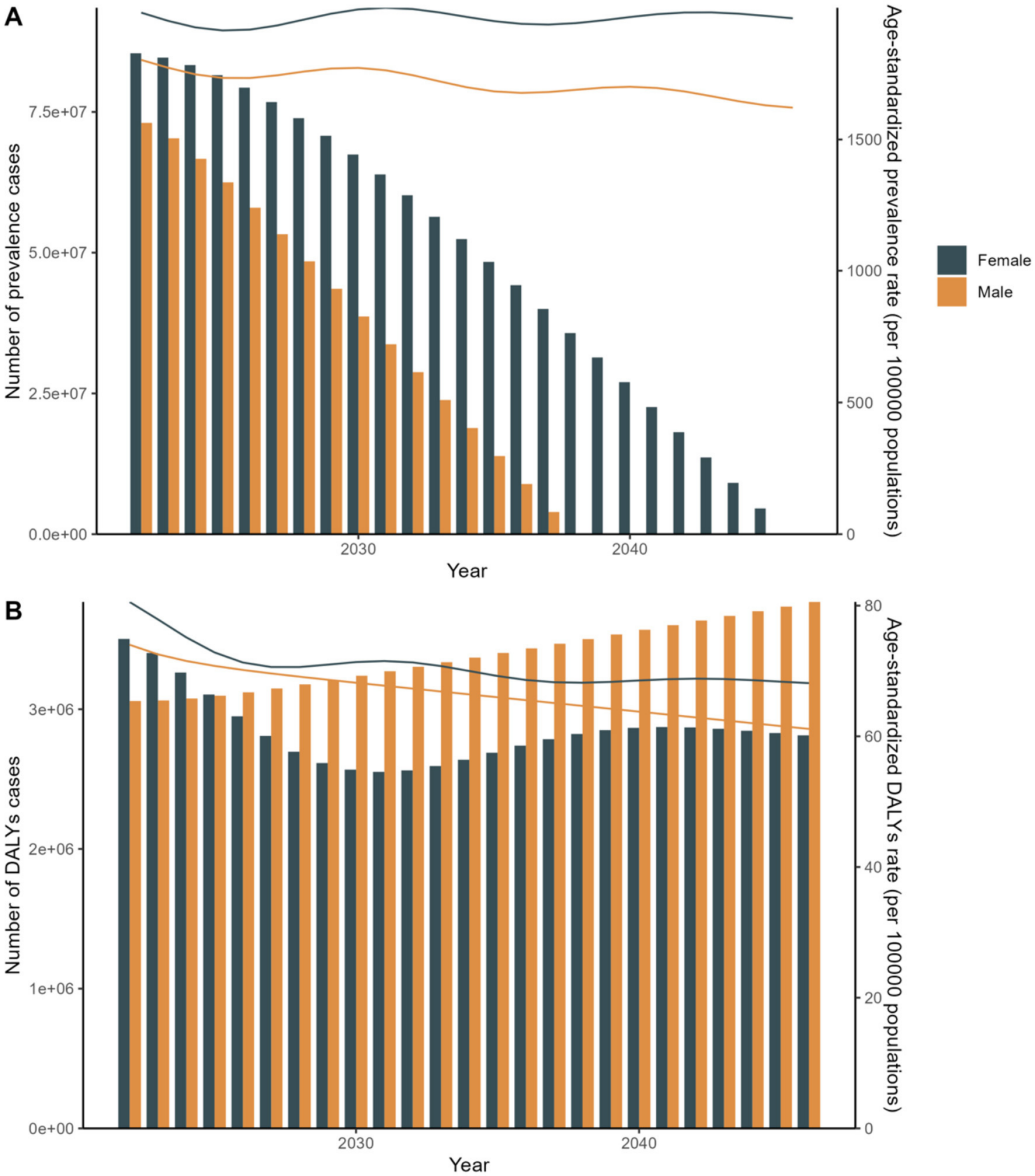
Arima: Linear regression analysis of the sex-specific age-standardized incidence rate for refraction disorders from 2030 to 2040. **(A)** Prevalence rate. **(B)** DALYs rate.

To validate the results of the APC analysis, we employed the ARIMA predictive model as a sensitivity analysis. In alignment with the APC analysis, the ARIMA predictive model reveals that from 1990 to 2049, women have a higher priority in terms of age-standardized incidence. These two models are essential for predicting future changes in disease prevalence trends and DALYs, and for optimizing public health policy and resource allocation. Both models offer valuable insights but from different analytical perspectives. The APC model is more suited for understanding the underlying factors driving trends, while the ARIMA model excels in robust time series forecasting. The choice of model depends on the specific research objectives and the type of insights sought. These conclusions underscore the importance of model selection in epidemiological forecasting and health policy planning, as different models can provide varied insights into future disease trends and burdens (Figure 12 and Appendix 4).

## 4 Discussion

To our knowledge, this study presents the most comprehensive and detailed analysis of the prevalence and burden of refractive disorders, leveraging the latest GBD 2021 data. The estimates derived from the GBD 2021 represent a significant advancement over previous iterations, attributable to the incorporation of novel data from diverse geographical locations (14).

### 4.1 From sex and age

Between 1990 and 2019, despite an increase in the total number of DALYs and stability in crude DALYs rates, age-standardized DALYs rates globally exhibited a downward trend. Our findings indicate that the global health burden of refractive disorders intensifies with age, is disproportionately higher among females, and is particularly significant in populations with lower socioeconomic status. We emphasize that women, particularly in low-income regions, will continue to constitute a larger proportion of those affected by refractive errors in the future. While our study does not differentiate between specific types of refractive errors [such as myopia (22), hyperopia (23), and astigmatism (24)], it underscores the critical need to improve access to medical resources for addressing refractive issues among women in economically disadvantaged areas. Additionally, our research reveals a notable prevalence of refractive errors among the older adult, often associated with age-related conditions like cataracts and macular degeneration (25).

This demographic trend reflects the growing burden of an aging population on healthcare systems (26). To address these challenges effectively, we recommend establishing comprehensive healthcare frameworks that encompass both psychological and physiological support systems tailored to the needs of older adult individuals with refractive errors. These findings contribute to a deeper understanding of refractive error epidemiology and advocate for targeted healthcare strategies that prioritize vulnerable populations, including women in low-income regions and the older adult facing age-related vision challenges. Visual impairment has been associated with a reduction in physical activity, and loss of vision significantly impacts the ability to perform basic activities of daily living, such as eating, dressing, writing, and even simple communication (27). Additionally, effective physical activity requires proper visual function, including visual acuity, contrast sensitivity, and a broad visual field, all of which can be compromised by various ocular pathologies (28). It is also important to consider cultural and social differences, which can influence how individuals experience and manage visual impairment (29). By recognizing these factors, our study underscores the necessity for comprehensive strategies that address not only the medical but also the social and cultural aspects of visual impairment. At the meantime, it is essential for developing effective interventions to maintain physical activities and overall well-being among those affected by visual impairment.

Retraction disorders significantly undermine not only educational prospects and overall quality of life but also leads to productivity losses, thereby contributing to the widening income gap between genders (30). Remarkably, Efforts to promote gender-inclusive data are hindered by its frequent absence in population-based surveys collecting health and gender indicators, highlighting the need for comprehensive strategies beyond data disaggregation alone to address health disparities among males, females, and gender-diverse individuals (31). Gender disparities in vision health are already apparent among individuals in their thirties. To achieve gender equality, as outlined in the Sustainable Development Goals, further research is imperative to analyze and address gender inequality over the next three decades. Targeted policies and strategic planning are essential to mitigate the specific risk behaviors, social dynamics, and healthcare access challenges faced by females and males across diverse regions (32). Without comprehensive and intersectional insights, the systemic barriers that perpetuate health inequities will persist unchallenged.

### 4.2 From country and region

Despite the traditional perception that refractive disorders are primarily a “China” condition, the GBD 2021 estimates present a contrasting global picture (33). Benin exhibited the largest increase in prevalence and burden, while India had the most significant decrease. Notably, the USA experienced the most significant increase in both prevalence and burden from 1990 to 2021. However, it is crucial to mention that the GBD estimates did not account for discrepancies in cases where survey informants disagreed. Consequently, the extent to which the observed differences in prevalence across countries reflect genuine disparities or are influenced by methodological factors remains uncertain.

To better understand these regional variations, it is essential to consider several underlying factors. One key factor is the accessibility of medical resources. In regions with limited access to ophthalmic care, such as Benin and other West African countries (34, 35), individuals may face barriers to accessing timely and appropriate treatment for refractive disorders. This can lead to increased prevalence and burden of refractive disorders in these areas. Conversely, in regions with well-established healthcare systems and widespread access to ophthalmic care, such as India, the implementation of preventive measures and early interventions may contribute to decreased prevalence and burden (36, 37).

Cultural customs also play a significant role in shaping regional differences in refractive disorders. In some regions, cultural beliefs or practices may affect individuals' willingness to seek medical treatment for refractive errors. For example, in certain communities, there may be a stigma associated with wearing glasses or seeking ophthalmic care, which can lead to delayed diagnosis and treatment. Additionally, variations in educational attainment and literacy levels across regions may influence individuals' understanding of refractive disorders and their importance in seeking appropriate care.

Environmental factors, such as urbanization and pollution levels, could also contribute to regional disparities in refractive disorders. In urban environments, high levels of air pollution and increased digital screen usage may increase the risk of refractive errors. This is particularly relevant in regions like the USA, where the prevalence and burden of refractive disorders have increased significantly over the past few decades. In contrast, rural areas with lower pollution levels and less exposure to screens may have lower risks of refractive disorders. However, rural populations may also face barriers to accessing ophthalmic care due to geographical isolation or limited healthcare infrastructure.

Given the increasing prevalence of refractive disorders and the associated socioeconomic costs, a comprehensive set of preventive measures is essential. We advocate for a multinational, integrated approach to prevention and control, leveraging the capabilities of the digital age to establish cross-regional, cross-national, and cross-temporal strategies for managing refractive disorders. This collaborative effort should include efforts to improve medical resource accessibility, address cultural barriers to care, and mitigate environmental factors that contribute to refractive disorders. By addressing these underlying factors, we can work toward reducing the global burden of refractive disorders and improving ocular health outcomes for all individuals.

### 4.3 From SDI

The distribution of per million inhabitants exhibits a substantial variation in accordance with economic development, ranging from 3.7 per million in low-income nations to 76.2 per million in high-income countries. However, socioeconomic status alone does not entirely determine the health burden imposed by refractive disorders. The quality of healthcare services is equally pivotal, as these conditions can remain undetected even in developed countries, likely due to ambiguities in the definition of refractive disorders (38). This implies that other factors, including race (39), cultural (11), and accessibility to ophthalmic services (40), also contribute to the overall health burden. It is crucial to mention that myopia imposes the heaviest burden among refractive disorders (41). Factors such as excessive ocular strain, improper reading posture, and prolonged exposure to inadequate lighting are significant contributors to myopia (42), particularly among younger generations in East Asia (43).

Furthermore, SDI serves as a dynamic gauge of a nation's socioeconomic advancement, reflecting changes influenced by various factors such as cultural traditions, governmental policies, and economic conditions (44). In regions with higher SDI values, cultural norms often prioritize outdoor activities, potentially linked to lifestyle choices, educational frameworks, and environmental considerations. However, the landscape has shifted notably since the onset of the COVID-19 pandemic in 2019, marked by a surge in digital engagement facilitated by platforms like TikTok (45). Certainly, the study argues that the narrowing of the urban-rural divide reflects the changing environment in rural areas, where an increasing number of digital devices are encouraging people to work closer to home and spend time indoors (46). This proliferation of electronic media has compressed individuals' outdoor pursuits, despite the known protective effects of extended outdoor activities on vision health (47).

Since 2019, refractive disorders problems have significantly increased, even in regions traditionally scoring high on the SDI. Although we have proposed solutions for managing prolonged screen time—such as maintaining sufficient viewing distance (48), proper workspace lighting (49), and using anti-glare glasses—the increased use of electronic screens (50), while seemingly enhancing social connectivity, has inadvertently hindered face-to-face emotional interactions. Addressing the physiological and psychological challenges posed by this social activity necessitates strategies that not only reduce the use of electronic devices but also promote interpersonal interactions outside the digital realm to ensure comprehensive eye health protection. Therefore, we advocate for broader social activities, both indoors and outdoors, as an urgent priority.

### 4.4 Predictive model analysis

Our research, aligned with APC and ARIMA analyses, reveals that from 1990 to 2049, women exhibit a higher age-standardized incidence of refractive errors. The ARIMA model predicts a future decline in age-standardized DALYs rates and prevalence by 2040, despite sustained high female representation. This decline may be influenced by increased global awareness due to economic development, enhanced education access, lifestyle changes, and improved healthcare systems supporting comprehensive vision care initiatives (51). Significant gender differences in refractive error prevalence are evident in our study, influenced by behavioral factors, hormonal fluctuations, healthcare access disparities, and genetic predispositions (52). These findings underscore the need for tailored public health strategies that consider both economic development impacts on health awareness and gender-specific determinants affecting eye health.

Understanding both the broader impact of economic development on public health awareness and the specific gender-related determinants of refractive errors underscores the importance of tailored public health strategies that address both economic and gender-specific factors influencing eye health.

## 5 Limitation

In considering the findings of our study, it is imperative to acknowledge its limitations. Firstly, our research inherits the well-documented general limitations of the GBD framework. While the GBD endeavors to mitigate biases inherent in self-reported data, such as recall bias and desirability bias, as well as non-sampling errors and differential diagnostic patterns, we acknowledge that the GBD methods cannot comprehensively address and correct for all biases in reported data or fully account for disparities in diagnostic likelihood for specific conditions. This is a recognized limitation of the GBD approach. Furthermore, variations in the criteria for defining refractive disorders among different countries have introduced challenges to this study. For instance, many studies on childhood refractive errors rely heavily on spherical equivalent (SE) methods, which may underestimate hyperopia rates due to the halving of positive cylinder power. This issue has been raised in previous literature (53), casting doubt on the accuracy of SE in assessing the prevalence and severity of refractive errors. These findings emphasize the importance of accounting for astigmatism levels when interpreting SE data on myopia and hyperopia rates in international comparisons, as criteria for defining these conditions can differ significantly across different populations and regions. We recognize that these limitations may impact the interpretability and generalizability of our findings (54). However, we believe that our study still provides valuable insights into the global trends in refractive disorders and highlights areas for future research and improvement.

Secondly, while the APC model aids in analyzing age, period, and cohort effects, its linear dependence issue persists despite using the intrinsic estimator method, potentially hampering accurate effect separation. Additionally, the ARIMA model's future trend predictions, based on historical data similarity assumptions, may overlook public health emergencies and medical technology advancements, leading to potential prediction inaccuracies.

Thirdly, the GBD study may be limited by inadequate adjustment for biases in small community samples and covariates related to informant agreement or diagnostic impairment, which could potentially interfere with the accurate assessment of the relationship between refractive disorders and socioeconomic factors.

Fourthly, the study did not differentiate between specific types of refractive disorders, such as myopia, hyperopia, and astigmatism, limiting our understanding of their prevalence characteristics, disease burden differences, and influencing factors. This may hinder the development of targeted prevention and control strategies.

Finally, the study did not fully explore interactions between socioeconomic, lifestyle, and genetic factors, which may jointly affect refractive disorders. This limits our comprehensive understanding of their etiology and mechanism.

## 6 Conclusion

In conclusion, our current study provides insights into the prevalence and DALYs of refractive disorders both locally and globally. Additionally, our findings suggest that the GBD 2021 may have underestimated the prevalence and burden of these disorders. Our recommendations focus on three key areas for future interventions: first, prioritizing dynamic interventions tailored to female and older adult populations. Second, emphasizing the adjustment of psychophysiological aspects in social life. Third, advocating for the global integration of prevention and control measures by drawing insights from diverse regions and countries. Contemporary people already face a lot of overspending, so don't overspend on your eye health. Given the profound impact of the GBD in guiding research and policymaking, the limitations discussed herein should be addressed in its subsequent editions.

## Author's note

Data pertaining to the disease burden of refractive disorders are of paramount importance for clinicians, patients, and stakeholders. Herein, we present global and country-specific estimates of the prevalence and DALYs of refractive disorders from 1990 to 2021, derived from the Global Burden of Disease (GBD) study.

## Data Availability

The original contributions presented in the study are included in the article/Supplementary material, further inquiries can be directed to the corresponding author.
